# Relationship between estimated glomerular filtration rate and outcome of ischemic stroke patients after mechanical thrombectomy

**DOI:** 10.1111/cns.13700

**Published:** 2021-07-13

**Authors:** Zhelv Yao, Hengheng Xu, Yue Cheng, Yun Xu

**Affiliations:** ^1^ Department of Neurology Affiliated Drum Tower Hospital Nanjing University Medical School Nanjing China; ^2^ Jiangsu Key Laboratory for Molecular Medicine Nanjing University Medical School Nanjing China; ^3^ Nanjing Medicine Center for Neurological and Psychiatric Diseases Nanjing China

**Keywords:** acute ischemic stroke, mechanical thrombectomy, renal function, time window

## Abstract

**Aims:**

We aimed to determine whether preprocedural renal function affects the outcome of acute ischemic stroke (AIS) patients with mechanical thrombectomy (MT) and whether this effect is modified by the onset‐to‐reperfusion time (OTR) and mediated by collateral status.

**Methods:**

Eligible patients with anterior circulation large vessel occlusion (LVO) who underwent MT between August 2018 and August 2020 were reviewed. The main outcome was good functional outcome [defined as modified Rankin Scale (mRS) of 0–2] at 3 months. Multivariable logistic regression analyses were conducted to explore the relationship between renal function and good functional outcome. A moderation analysis and the Johnson‐Neyman technique were performed to assess the interaction between renal function and OTR to predict the outcome of AIS patients with MT.

**Results:**

Among 100 enrolled patients, 36 (36%) exhibited good functional outcome. A decreased preprocedural estimated glomerular filtration rate (pre‐eGFR) was significantly associated with worse functional outcome [adjusted OR, 1.059 (1.012–1.108); *p*, 0.014], and this effect was partly mediated by collateral circulation. An interaction between OTR and pre‐eGFR on functional outcome was observed (P for interaction, 0.22), and pre‐eGFR only had a significant effect on functional outcome when OTR exceeded 455.8 min. Moreover, the adverse effect of OTR on functional outcome became no longer significant when the pre‐eGFR was higher than 89.0 mL/min/1.73 mL/min/1.73 m².

**Conclusions:**

Renal function was related to functional outcome at 3 months, and this relationship could be modified by OTR. The results suggested that reducing OTR and improving collateral circulation may mitigate the adverse effect of reduced kidney function on functional outcome.

## Graphical abstract

The relationship between preoperative estimated glomerular filtration rate and functional outcome varies according to the onset‐to‐reperfusion time, reducing OTR may mitigate the negative effect of renal dysfunction.

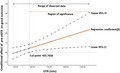

## 1 INTRODUCTION

Mechanical thrombectomy (MT) is a mainstay in acute stroke treatment and benefits patients with acute ischemic stroke with large vessel occlusion (LVO).^1^ However, not all patients have a good outcome following MT. It is necessary to have a deeper understanding of the factors associated with the outcome after MT to more accurately predict the benefits to individual patients and help clinics in decision‐making regarding whether to perform MT.

Renal impairment, characterized by a reduced estimated glomerular filtration rate (eGFR), is prevalent in patients with acute ischemic stroke (AIS). Recently, two studies demonstrated that renal dysfunction was an independent predictor of the functional outcome for patients treated with MT.^2^, ^3^ However, Laible et al. found that reduced eGFR was not associated with poor functional outcome of AIS patients receiving MT.^4^ The relationship between renal function and functional outcome may be affected by other factors.

The onset‐to‐reperfusion time (OTR) is generally considered a critical important determinant of functional outcome in patients receiving MT, and every 30‐minute delay may yield a 12–21% decrease in the likelihood of regaining functional independence.^5^ Given the growing evidence on the impact of renal function on cerebral vascular structure^6^, ^7^ and hemodynamics,^8^, ^9^ patients with worse renal function would be expected to be more vulnerable to ischemic status, which might be related to the rapid evolution of ischemia to infarction. From this perspective, patients with worse renal function would need to acquire successful recanalization within a shorter time than patients with normal renal function to salvage the function of the ischemic penumbra to achieve similar functional outcomes. The impact of OTR on functional outcome might also differ according to renal function.

It is well known that collateral circulation is related to the clinical and radiological outcomes of AIS patients.^10^, ^11^ Collateral circulation is an established key factor determining the pace of evolution of ischemic penumbra into the ischemic core.^12^, ^13^ Similar anatomical and functional characteristics of vessels have been shown in the kidney and brain, and some shared vascular risk factors have been found in renal dysfunction and AIS. Whether cerebral collateral circulation affects the relationship between renal function and MT outcome needs to be studied.

In the present study, AIS patients receiving MT between August 2018 and August 2020 at our stroke center were included. We investigated the relationship between renal function and functional outcome at 3 months and the potential effects of collateral circulation and OTR on the relationship. We hypothesized that renal function was independently associated with functional outcome following MT, and this link was partially mediated by collateral flow and could be modified by OTR.

## 2 METHODS

### 2.1 Study population

We performed a retrospective analysis of a prospective registry of consecutive AIS patients who underwent MT for anterior circulation LVOs at our stroke center between August 2018 and August 2020. The patients were deemed candidates for MT according to national guideline recommendation and neuro‐interventionist's evaluation. They also received standard treatment for AIS. During this period, patients were considered eligible for this study if they met the following criteria: modified Rankin Scale (mRS) of 0 or 1 before stroke; age ≥ 18 years; treatment initiated (ie, onset‐to‐puncture) within 24 h of symptom onset. Study criteria further required availability of serum creatinine values on admission. Participants were excluded if they lack of computed tomographic angiography (CTA) image or the quality of CTA image was poor due to motion artifact. This research was approved by the Ethics Committee of Nanjing Drum Tower Hospital, and the individual informed consent was waived.

### 2.2 Data collection and assessment

Information, such as demographics, vascular risk factors, admission serum creatinine, admission blood pressure, treatment parameters [OTR, modified Thrombolysis in Cerebral Infarction (mTICI) grade], LVO location, and image data [collateral status, Alberta Stroke Program Early CT Score (ASPECTS)], was collected from our electronic database (see Table 1 for details). OTR refers to the time to recanalization during the MT, or the end of the procedure for those without reperfusion.^14^ Preprocedural estimated glomerular filtration rate (pre‐eGFR) was calculated by using creatinine‐based Chronic Kidney Disease Epidemiology (CKD‐EPI) equation^15^; successful recanalization was defined as TICI scores 2b and 3.

**TABLE 1:** Comparisons of baseline characteristics in study population with poor and good outcome

	All patients (n = 100)	Good outcome (n = 36)	Poor outcome (n = 64)	*p*
Age, y, mean (SD)	70.7 (11.7)	67.0 (12.6)	72.8 (10.7)	0.017*
Male, n(%)	54 (54.0)	19 (52.8)	35 (54.7)	0.854
Initial NIHSS score, median (IQR)	15 (11–18)	11 (10–15)	16 (13–20)	<0.001***
ASPECTS, median (IQR)	8 (7–10)	9 (8–10)	8 (7–10)	0.316
Good collateral status, n (%)	39 (39.0)	25 (69.4)	14 (21.9)	<0.001***
mTICI(2b‐3), n (%)	87 (87.0)	34 (94.4)	53 (82.8)	0.177
OTR, min; median (IQR)	455.0 (320.0–660.0)	480.0 (325.0–620.0)	445.0 (310.0–710.0)	0.954
Thrombolysis with rtPA, n (%)	38 (38.0)	15 (41.7)	23 (35.9)	0.571
Site of occlusion, n (%)				
ICA	31 (31.0)	8 (22.2)	23 (35.9)	0.159
M1 MCA	50 (50.0)	18 (50.0)	32 (50.0)	
M2 MCA or other tributaries	19 (19.0)	10 (27.8)	9 (14.1)	
History of TIA or stroke, n (%)	19 (19.0)	5 (13.9)	14 (21.9)	0.328
Coronary disease, n (%)	13 (13.0)	2 (5.6)	11 (17.2)	0.177
Hypertension, n (%)	67 (67.0)	19 (52.8)	48 (75.0)	0.023*
Diabetes, n (%)	27 (27.0)	3 (8.3)	24 (37.5)	0.002**
Dyslipidemia, n (%)	30 (30.0)	13 (36.1)	17 (26.6)	0.317
Atrial fibrillation, n (%)	56 (56.0)	18 (50.0)	38 (59.4)	0.365
Habitual smoking, n (%)	20(20.0)	8(22.2)	12(18.8)	0.677
Alcohol assumption, n (%)	18(18.0)	9(25.0)	9(14.1)	0.172
Baseline SBP, mmHg; mean (SD)	138.0 (20.4)	138.9 (20.3)	137.5 (20.5)	0.744
Baseline DBP, mmHg; mean (SD)	76.8(14.3)	77.1 (15.2)	76.7 (13.9)	0.888
Pre‐eGFR, mL/min/1.73 m ²; mean (SD)	81.3 (22.6)	94.0 (17.4)	74.2 (22.2)	<0.001***

Abbreviations: ASPECTS, Alberta Stroke Program Early CT Score; DBP, diastolic blood pressure; ICA, internal carotid artery; IQR, interquartile range; MCA, middle cerebral artery; mTICI, modified Thrombolysis in Cerebral Infarction; NIHSS, National Institutes of Health Stroke Scale; OTR, onset‐to‐reperfusion time; pre‐eGFR, preoperative estimated glomerular filtration rate; rtPA, tissue‐type plasminogen activator; SBP, systolic blood pressure; SD, standard deviation.

* *p *< 0.05, **p < 0.005, ***p < 0.001.

### 2.3 Imaging analysis

All participants had a multimodal stroke imaging, including non‐contrast head CT and CT angiography (CTA) performed in the emergency room, and all CT sequences were acquired on a 64‐slice CT device (Discovery CT750 HD, GE Healthcare, Milwaukee, WI, USA). 3D orthogonal maximum intensity projection (MIP) images were created in three planes. Collaterals were evaluated on MIP images of CTA by two experienced neuroradiologists using the Tan score: 0 = no collaterals visible; 1 = diminished collaterals in >50% of the occluded arterial territory; 2 = absent collaterals in <50% of the occluded territory; and 3 = collateral equal to the contralateral normal hemisphere.^16^ For analysis purpose, the scores were dichotomized into either good collaterals (grades 2 and 3) or poor collaterals (grades 0 and 1).

### 2.4 Outcome measures

Our main outcome was functional status, assessed at 3 months by trained staff via phone interview using a modified Rankin Scale (mRS) questionnaire. Functional independence (ie, good functional outcome) was defined as mRS 0‐2, while poor functional outcome was defined as an mRS score 3 or higher.

### 2.5 Statistical analyses

Patients were dichotomized based on their clinical outcome using mRS. Data were expressed as mean (SD), median (interquartile range), or number (percentage), as appropriate. Univariable comparison of good and poor outcome patients in baseline demographic, clinical, and procedural variables was performed using Student’ s *t* test or Mann–Whitney *U* test for metric variables and chi‐square or Fisher's exact test for qualitative variables, as appropriate. Kolmogorov‐Smirnov tests were applied to test the normality of variables.

There are three main statistical steps. First, to test the association between pre‐eGFR and functional outcome, the multivariable regression model was established based on *p *< 0.1 in the univariate analysis plus the variables that were generally considered to be associated with functional outcome in prior literature: age, diabetes, hypertension, collateral status, NIHSS, gender, TICI grade, ASPECT, and OTR. Covariates were assessed for interaction effects. Multicollinearity was checked by variance inflation factor (VIF) tests. All VIF values were less than 1.746, which implies absence of severe multicollinearity.^17^ When pre‐eGFR was a significant predictor, the optimal sensitivity‐specificity cutoff point of pre‐eGFR associated with good functional outcome was calculated using receiver operating characteristic curve (ROC) analysis.

Subsequently, we explored the mediating role of collateral status on the relationship between pre‐eGFR and good functional outcome at 3 months. Given that both the dependent variable and mediator were categorical, we adopted the statistical strategy for assessing mediating effects of categorical variables via Sobel test proposed by Iacobucci,^18^ which comprises three separated logistic regression equations. In the first regression equation, the mediator (ie, collateral status) was regressed on the independent variable (ie, pre‐eGFR) by logistic regression analysis. In the next regression equation, the dependent variable (ie, good functional outcome) was regressed on the independent variable (ie, pre‐eGFR) by logistic regression analysis. In the last regression equation, the dependent variable (ie, good functional outcome) was regressed on both the independent variable (ie, pre‐eGFR) and the mediator (ie, collateral status). All the three equations were adjusted for the following potential confounders: age, baseline NIHSS score, ASPECT, diabetes, hypertension, gender, OTR, and TICI grade. We also include OTR×pre‐eGFR interaction as a covariate term. The test is significant at α = 0.05, if Zmediation > |1.96|. Stata's binary_mediation command was utilized to calculate the indirect effect.

Third, the hypothesis that the effect of pre‐eGFR would be different by OTR was tested using Model 1 in PROCESS macro 3.0 for IBM SPSS. A bias‐corrected bootstrap method with 5000 bootstrapping samples was utilized. The multivariable logistic regression model mentioned above was generated to elucidate the interaction effect. When there existed interaction effect, the Johnson‐Neyman technique was further applied to identify specific OTR in which the significant association between the effects of pre‐eGFR, functional outcome appears or disappears, and how this relationship varies based on OTR.^19^

All *p* values presented were two‐tailed, with values <0.05 (less than 0.10 for interaction terms) defining statistical significance.^20^ All statistical analyses were completed using SPSS software (IBM, Chicago, IL, USA), version 25.0, and Stata (StataCorp LP, College Station, TX, USA), version 16.0.

## 3 RESULTS

### 3.1 Demographic and clinical characteristics

Of the 126 patients with anterior LVO stroke receiving MT enrolled from August 2018 to August 2020, 105 patients had baseline serum creatinine and CTA, five patients were subsequently excluded for initial mRS score of 3 or greater (n = 2), lost of follow‐up (n = 2), and presence of motion artifacts (n = 1), leaving 100 subjects for the final analyses. Table 1 displays the characteristics of included patients according to dichotomized mRS score of 0–2 or greater. Across the entire study population, the mean age was 70.7±11.7 years, 54 patients (54.0%) were male, mean pre‐eGFR was 81.3 mL/min/1.73 mL/min/1.73 m² (range, 24.0–128.8 mL/min/1.73 m²), and median NIHSS score was 15 (IQR 11–18). Eighty‐seven subjects (87.0%) showed recanalization success and 36 subjects (36.0%) achieved good outcome at 3 months. The median OTR of the overall population was 455.0 min (range, 210–1350 min).

As shown in Table 1, patients with good outcome were younger (mean 67.0 versus 72.8 years, *p *= 0.017), had a higher mean pre‐eGFR (94.0 vs 74.2 mL/min/1.73 mL/min/1.73 m², *p *< 0.001) and a lower median NIHSS score (11 vs 16, *p *< 0.001), and had better collateral status compared to patients with poor outcome (69.4% vs 21.9%, *p *< 0.001). Hypertension (52.8% vs 75.0%, *p *= 0.023) and diabetes mellitus (8.3% vs 37.5%, *p *= 0.002) were less commonly found in patients with good outcome. No significant difference in the site of occlusion, rates of hyperlipidemia, blood pressure on admission, and tissue‐type plasminogen activator (rtPA) use was shown between the two groups.

### 3.2 Prediction of good outcome at 3 months

The NIHSS, diabetes, collateral status, and pre‐eGFR were significantly related to 90‐day outcome and remained significant in multivariable logistic regression (Table 2). Higher pre‐eGFR (OR 1.059; 95% CI, 1.012–1.108; *p*, 0.014) and a better collateral circulation (OR 10.138; 95% CI, 2.296–44.761; *p*, 0.002) significantly increased the likelihood for good outcome. Both history of diabetes (OR 0.059; 95% CI, 0.008–0.434; *p*, 0.005) and increasing NIHSS (per 1‐point increase; OR 0.836; 95% CI, 0.717–0.975; *p*, 0.023) reduced the likelihood for good outcome. The ROC curve analysis indicated that pre‐eGFR predicting outcome was significant. The area under the ROC curve was 0.761 (95% CI: 0.665–0.856, *p *< 0.001), indicating good predictive accuracy.^21^ The best cutoff value of the pre‐eGFR predict good outcome was preGFR ≥ 86.55 mL/min/1.73 m², yielding a sensitivity of 69.4% and a specificity of 71.9%.

**TABLE 2:** Prediction of good functional outcome (mRS 0‐2)

Independent variable	Model I		Model II	
	Odds ratio (95% CI)	*p*	Odds ratio (95% CI)	*p*
Collateral status	8.117 (3.221–20.453)	<0.001***	10.138 (2.296–44.761)	0.002**
NIHSS	0.833 (0.752–0.923)	<0.001***	0.836 (0.717–0.975)	0.023*
Diabetes	0.152 (0.042–0.548)	0.004**	0.059 (0.008–0.434)	0.005*
Pre‐eGFR	1.049 (1.024–1.075)	<0.001***	1.059 (1.012–1.108)	0.014*
HBP	0.373 (0.157–0.885)	0.025*	0.666 (0.174–2.554)	0.553
Age	0.957 (0.922–0.993)	0.021*	0.984 (0.915–1.058)	0.661
OTR	1.000 (0.999–1.002)	0.641	0.995 (0.991–0.999)	0.017*

Note

Model Ⅰ: unadjusted.

Model Ⅱ: adjusted for variables with *p* < 0.1 in univariate analysis plus gender, TICI grade, ASPECT, OTR and OTR×pre‐eGFR.

NIHSS indicates National Institutes of Health Stroke Scale; pre‐eGFR, preoperative estimated glomerular filtration rate.

* *p *< 0.05, **p < 0.005, ***p < 0.001.

We found that OTR turned out to be an independent factor related to functional outcome when it entered the multivariable model. Every 30‐minute delay in OTR corresponded to a 15% relative reduction in the possibilty of regaining a favorable outcome at three months (OR 0.995; 95% CI 0.991–0.999; *p*, 0.017).

### 3.3 Collateral circulation on the association between pre‐eGFR and functional outcome

**FIGURE 1:**
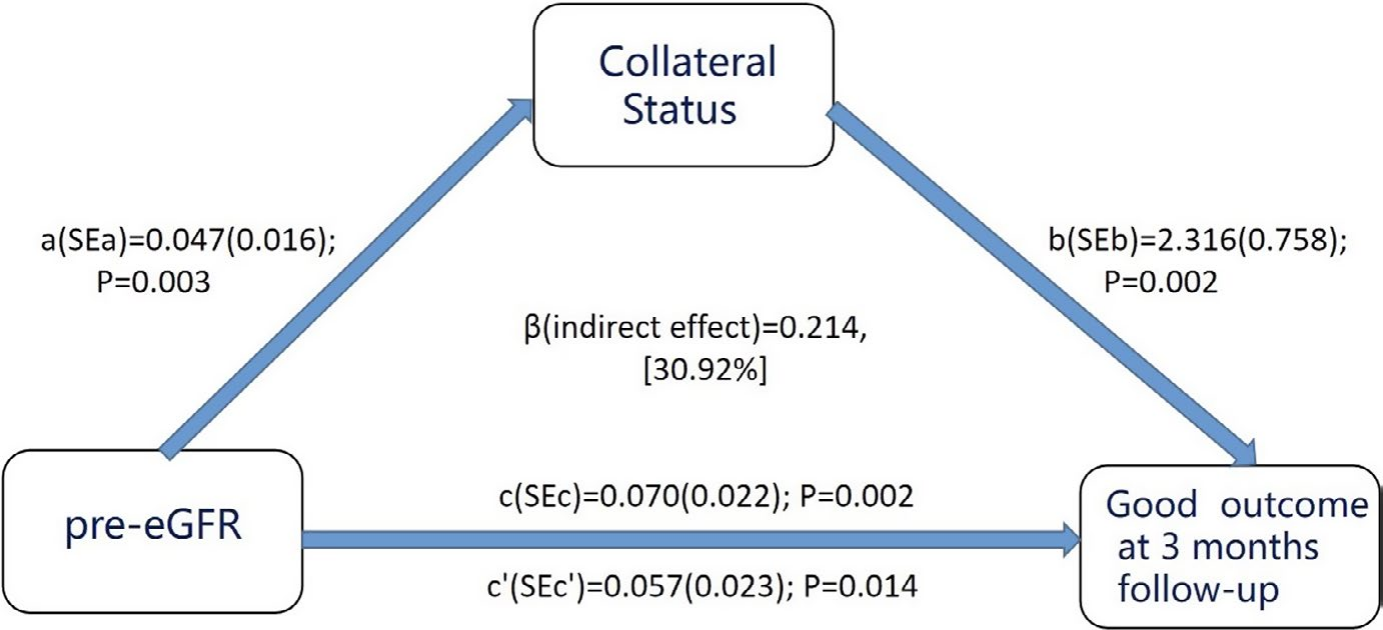
Mediation analysis. Mediation model displaying standardized path coefficients between pre‐eGFR, collateral status, and good outcome at 3 months, controlling for potential covariates (namely, age, baseline NIHSS score, diabetes, hypertension, gender, TICI grade, ASPECT, OTR, and OTR×pre‐eGFR). Note: The path from pre‐eGFR to good outcome at 3 months includes the coefficient with adjustment for collateral status (c′) and without adjustment for collateral status (c). All individual pathways were satisfied

Mediation analyses were performed to estimate the direct and indirect association between pre‐eGFR and good outcome at 3‐month follow‐up, with the collateral status as a proposed mediator. The collateral status significantly mediated the association of pre‐eGFR with good outcome, and the percentage of mediated effect was 30.92% (Figure 1; Table 3).

**TABLE 3:** Mediation analysis

		Model Ⅰ	Model Ⅱ
Pre‐eGFR on collateral status	a (SEa)	0.036 (0.011)	0.047 (0.016)
	P	0.001**	0.003**
Collateral status on good outcome	b (SEb)	1.771 (0.500)	2.316 (0.758)
	P	<0.001***	0.002**
Mediated Effect	β	0.152	0.214
	Sobel test (Z)	2.404	2.118
	P	0.016*	0.034*
Proportion Mediated %		27.36	30.92

Note

Model Ⅰ: unadjusted

Model Ⅱ: adjusted for age, baseline NIHSS score, diabetes, hypertension, gender, TICI grade, ASPECT, OTR and OTR×pre‐eGFR

Pre‐eGFR indicates preoperative estimated glomerular filtration rate.

β indicates the standardized regression coefficient of indirect effect.

* *p *< 0.05, **p < 0.005, ***p < 0.001.

### 3.4 Interactions between pre‐eGFR and OTR in predicating functional outcomes

After adjusting for potential confounders, there was a significant interaction between pre‐eGFR and OTR (*p*_interaction_, 0.022; Table 4).

**TABLE 4:** Multivariable logistic regression analyses of the pre‐eGFR×OTR interaction association with the 90‐day functional outcome

Independent variable	Odds ratio (95% CI)	*p*
Model Ⅰ		
Pre‐eGFR	1.057 (1.026–1.090)	<0.001***
OTR	0.998 (0.995–1.001)	0.182
Pre‐eGFR ×OTR	1.000 (1.000–1.000)	0.092
Model Ⅱ		
Pre‐eGFR	1.059 (1.012–1.108)	0.014*
OTR	0.995 (0.991–0.999)	0.017*
Pre‐eGFR ×OTR	1.000 (1.000–1.000)	0.022*

Note

Model Ⅰ: unadjusted.

Model Ⅱ: adjusted for age, baseline NIHSS score, diabetes, hypertension, collateral status, gender, TICI grade, and ASPECT.

Pre‐eGFR indicates preoperative estimated glomerular filtration rate; and OTR, onset‐to‐reperfusion time.

* *p *< 0.05, **p < 0.005, ***p < 0.001.

**FIGURE 2:**
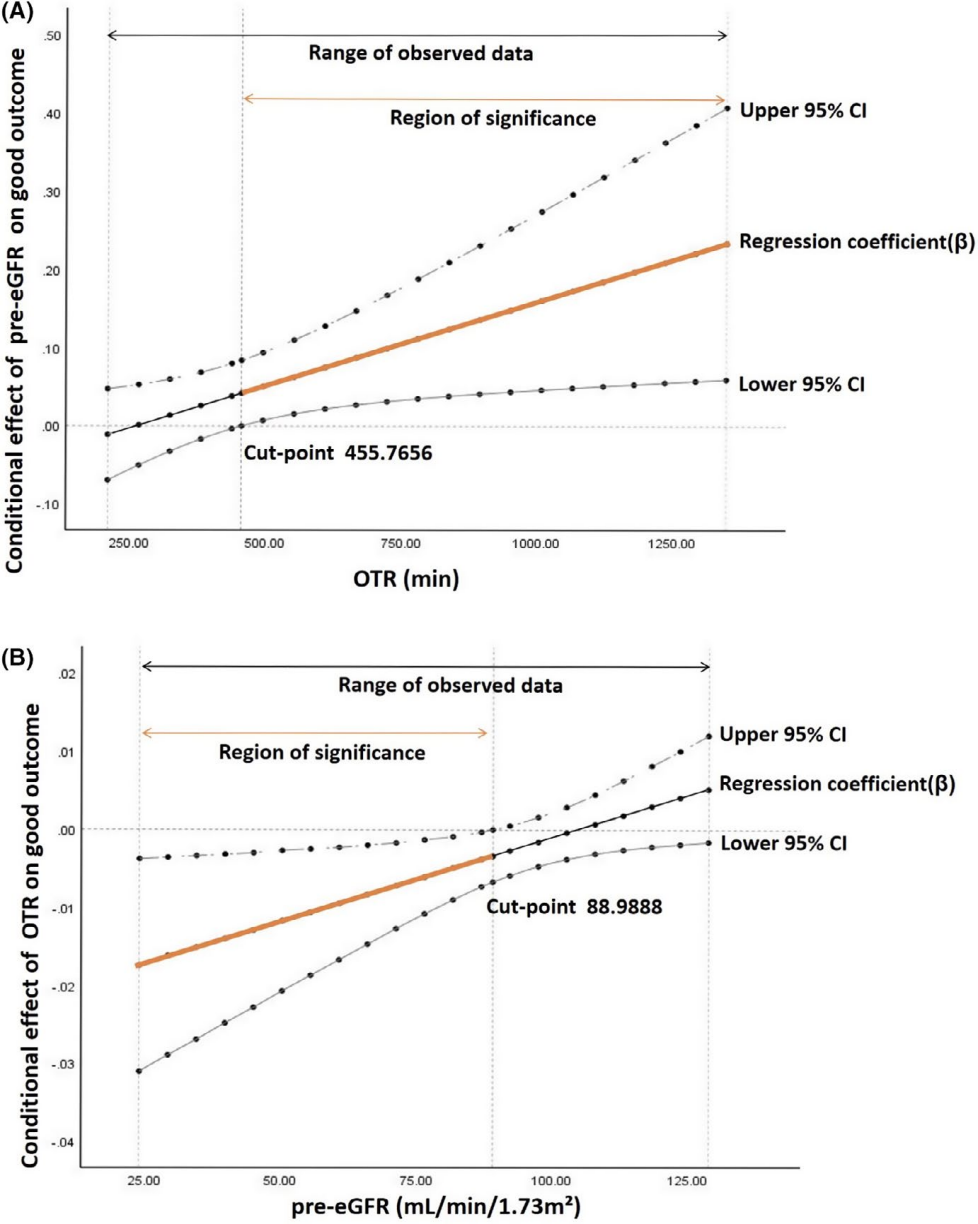
(A) The conditional effect of pre‐eGFR on functional outcome across the range of OTR. Regression slope evaluates the conditional effect of pre‐eGFR on functional outcome across OTR, based on Johnson‐Neyman analysis, adjusted for age, baseline NIHSS score, diabetes, hypertension, collateral status, gender, TICI grade, and ASPECT. The region of statistical significance lies in where the confidence band does not include the horizontal zero‐line. The vertical lines indicate the point at which the lower or upper confidence interval crosses the zero‐line. (B) The conditional effect of OTR on the outcome across the range of pre‐eGFR. Regression slope evaluates the conditional effect of OTR on the outcome across pre‐eGFR, based on Johnson‐Neyman analysis, adjusted for age, baseline NIHSS score, diabetes, hypertension, collateral circulation, gender, TICI grade. and ASPECT

Since there was evidence of OTR to pre‐eGFR interaction, we further quantified the interaction effect using the Johnson‐Neyman analysis. Figure 2A displays the results acquired from this analysis and presents the upper and lower confidence intervals of the slopes estimating the effect of pre‐eGFR on functional outcome for the range of OTR data from the entire sample. As Johnson‐Neyman technique presented, the relationship between pre‐eGFR and functional outcome was significant and positive for the substantial majority of the range of OTR in the current study, yet this association became weakened with the decrease in OTR. When OTR was less than 455.7656 min, the effect of pre‐eGFR on functional outcome was no longer substantial.

The moderating role of pre‐eGFR in the association between OTR and functional outcome is shown in Figure 2B. The range of pre‐eGFR is 24.0–128.8 mL/min/1.73 m² in the current sample. According to the range of simple slopes obtained from the Johnson‐Neyman analysis, the relationship between OTR and functional outcome was insignificant for pre‐eGFR 88.9888 mL/min/1.73 m² or higher. However, once the pre‐eGFR was lower than this threshold, shorter OTR was significantly associated with higher possibility of good outcome, and the association became stronger as the pre‐eGFR decreased.

## 4 DISCUSSION

Concordant with our hypothesis, our findings confirmed that preprocedural renal function was an independent determinant of functional outcome, and this association was partially mediated by collateral status. More importantly, there existed an interaction between pre‐eGFR and OTR on functional outcome, and the impact of renal function on functional outcome decreased with the shortening of OTR and was no longer substantial when OTR was less than 455.8 min; the adverse effect of long OTR on functional outcome declined progressively as pre‐eGFR increased and became insignificant when pre‐eGFR was higher than 89.0 mL/min/1.73 m².

Our results are consistent with previous findings of two cohort studies demonstrating that renal function is independently associated with functional outcome.^2^, ^3^ Notably, we extended prior knowledge of this association to a further step based on the mediation analysis, which suggested that collateral status partially mediated the impact of kidney function on functional outcome. Collateral status is a well‐established predictor of functional outcome.^22^ Similar anatomical and functional characteristics make the kidney and brain vulnerable to common vascular risk factors.^23^, ^24^, ^25^ Renal‐specific risk factors, such as chronic inflammation, anemia, and oxidative stress, as well as uremia‐related factors are also believed to contribute to cerebrovascular injury via effects on the arterial medial wall and endothelium. The vasodilatory capacity of the leptomeningeal collaterals is one of the potential mechanisms for collateral recruitment. Renal dysfunction possibly impairs endothelial function,^26^, ^27^ promotes atherosclerotic vessel stenosis and vascular calcification,^28^, ^29^ and consequently reduces the vasodilatory capacity of leptomeningeal collaterals in cases of ischemia. Patients with worse renal function are also found to have reduced production of nitric oxide^30^ and a decreased number and function of endothelial progenitor cells,^31^ which contribute to impaired collateral angiogenesis.

We also found that the effect of pre‐eGFR on functional outcome weakened with shorter OTR and disappeared in patients with OTR less than 455.8 min. In line with our study, Laible et al. conducted a cohort study (median onset‐to‐puncture time, 227 min) and reported that eGFR was not related to functional outcome.^4^ This time‐dependent phenomenon may be partly explained by the hypothesis that collateral flow requires a relatively longer time to develop and thus mediates the association between eGFR and functional outcome.^32^ Hence, it is conceivable to hypothesize that renal function may exert effects on outcome in a time‐dependent manner. Further studies specifically designed to address this issue are needed.

An additional novel finding of the current study is that the likelihood of a good outcome of MT declines at different paces with OTR according to renal function. Consistent with prior observations, our study observed an overall 15% decrease in the likelihood of achieving good outcomes for every 30‐minute delay. This relationship of OTR with functional outcome, however, weakened with increased eGFR and was no longer substantial when the pre‐eGFR passed the threshold of 89.0 mL/min/1.73 m². A potential mechanism would be the different underlying collateral flow patterns between patients with normal renal function and those with reduced kidney function.^22^ The quality of collateral circulation is generally recognized as a key factor determining the pace of penumbral tissue loss^12^, ^13^ and was revealed to be affected by renal function in our study. Reduced kidney function may damage collateral flow and subsequently accelerates the evolution of ischemia to infarction and lowers the odds of a good outcome. Therefore, the outcome of patients with reduced kidney function is more sensitive to OTR, whereas the outcome of those with normal renal function is relatively resistant to long OTR.

These findings provide a preliminary reference for the management of AIS patients, especially those with decreased pre‐eGFR. Given the huge pathological difference between patients with reduced kidney function and those with normal renal function, blindly extrapolating the results in the general population to these patients carries great risks. Considering that shortening the OTR can mitigate the negative effect of renal dysfunction, we recommend that patients with reduced baseline kidney function should achieve reperfusion in the fastest possible time. Furthermore, the findings derived from the mediation analysis suggested that collateral vessels can be considered a promising therapeutic target in patients with reduced kidney function. Additionally, as eGFR values are independently associated with functional outcome in patients undergoing MT, eGFR assessment prior to treatment may help in the prediction of prognoses.

Our findings also provide a rationale for selecting eligible patients for MT in an extended time window. Our results suggested that improving and streamlining workflows to reduce delays to endovascular therapy need to particularly focus on patients with reduced kidney function, and it is better to achieve successful reperfusion within 455.8 min. On the other hand, in patients with good renal function, the time window of benefit may be longer. However, our analysis did not imply that the time window can be extended indefinitely in these cases. As expedited therapy is always of great importance and our results are based on a tested timeframe of 24 hours, further exploration with a wider range of timeframes and larger series is warranted to better define the optimal cutoff time for patients with normal renal function.

Several potential limitations should also be noted. First, this is a study based on a single center. The generalizability of our findings should be tested in a larger sample from multicenters. Second, patients may have been selected for treatment in prior based on other favorable clinical and imaging features, which may affect the time effect expected in the general population. Lastly, our statistical adjustments for patient clinical differences may be incomplete because some unmeasured or residual confounding variables may exist. Therefore, our results should be interpreted with caution and await confirmation from a larger, prospective multicenter study.

## 5 CONCLUSIONS

To conclude, our study indicated that decreased pre‐eGFR is related to poor 90‐day functional outcomes in patients with anterior circulation LVO AIS with MT and can serve as a prognostic marker for selecting eligible patients for MT. Although all patients need to achieve recanalization as soon as possible, the time window may close earlier for patients with reduced kidney function than for patients with good renal function. These findings furthered the understanding of the relationship between renal function and the functional outcome of ischemic stroke patients receiving MT and may contribute to planning treatments in patients with decreased renal function.

## CONFLICTS OF INTEREST

The authors declare that they have no conflicts of interest.

## AUTHORS’ CONTRIBUTIONS

YX contributed to conceptualization; ZY and HX collected the data; ZY and YC made statistical analysis; ZY wrote original draft; YX reviewed and edited the manuscript; YX acquired funding; and YX supervised the study.

## Data Availability

Anonymous data that support the findings of this study are available on reasonable request from the corresponding author.
